# Supplementary figures and images for: Epilogos: information-theoretic navigation of multi-tissue functional genomic annotations
Source: bioRxiv. 2025 Jun 23:2025.06.18.660301. Preprint. [Version 1] doi: 10.1101/2025.06.18.660301 (PMC12262391; doi:10.1101/2025.06.18.660301)

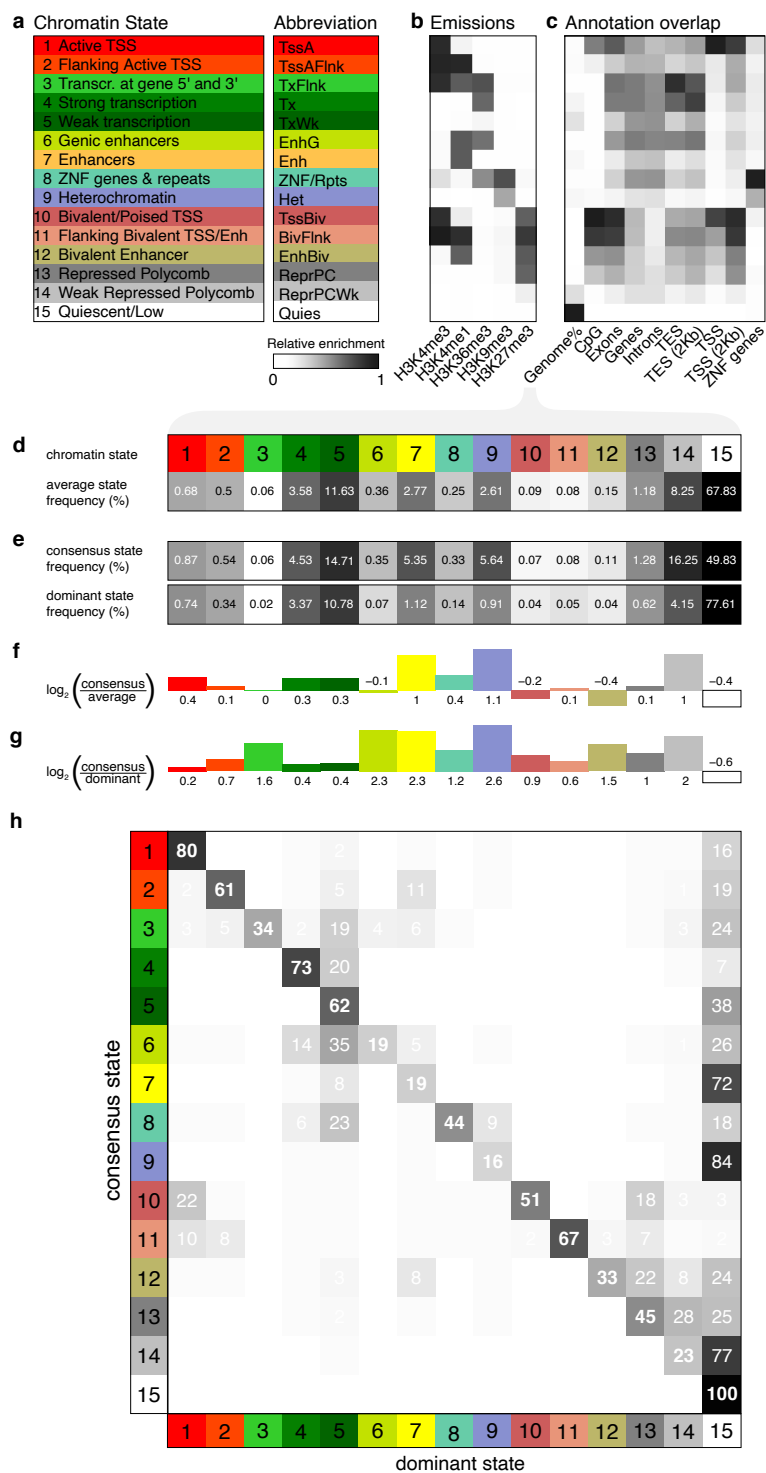

Supplement: Supplement 1 — Supp. Figure 1 – Chromatin state models and saliency scores (a-c) A 15-state ChromHMM model as used across 127 reference epigenomes12, based on H3K4me1, H3K4me3, H3K27me3, H3K36me3 & H3K9me3 histone tail modification ChIP-seq data. Shown are (a) chromatin state labels and abbreviations, (b) emission matrix containing the combinatorial patterns of epigenomic marks per chromatin state and (c) enrichments of various genomic annotations and functional activity levels per chromatin state. (d) Average genome-wide chromatin state frequencies across all 127 epigenomes, used as background (expected) frequencies for calculating Epilogos saliency scores. (e) Consensus state frequencies, as determined by per-position maximum saliency states, shown alongside state frequencies as determined by choosing the dominant state at each position. (f-g) Comparison of consensus versus (f) average and (g) dominant state frequencies, highlighting the over-representation of regulatory and other non-quiescent states in the consensus states. (h) Comparison of per-position consensus versus dominant state frequencies, highlighting an over-representation of quiescent state calls in the dominant state frequencies. [file media-1.pdf]

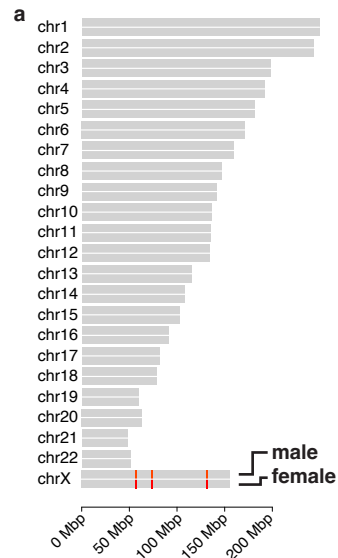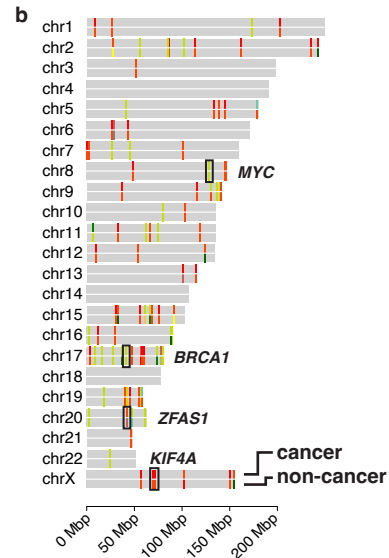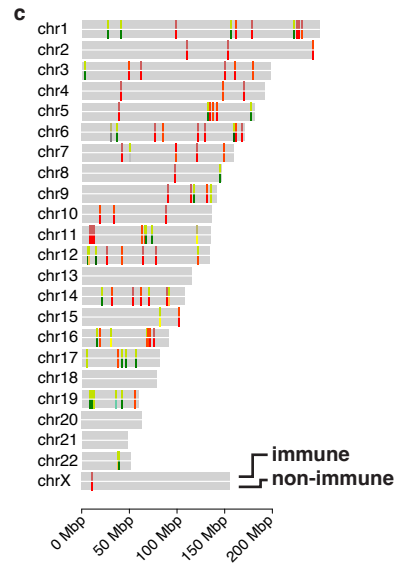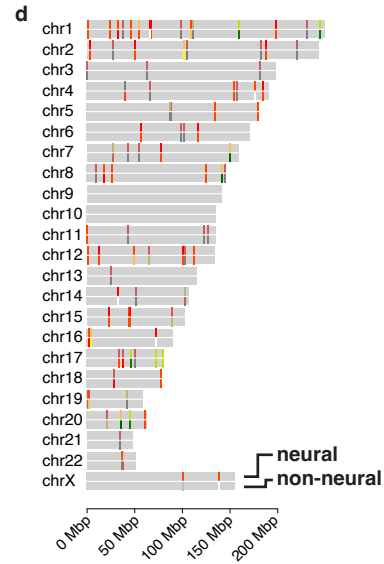

Supplement: Supplement 2 — Supp. Figure 2 – Top 100 differential regions for various groupings (a) Statistically significant differential regions between male and female donor biosamples, highlighting only X-inactivation related genes and regions on chromosome X. (b) Differential regions between cancer and non-cancer biosamples, highlighting cancer related genes BRCA1, ZFAS1, KIF4A, & MYC. (c) Differential regions between immune and non-immune biosamples. (d) Differential regions between neural and non-neural biosamples. [file media-2.pdf]

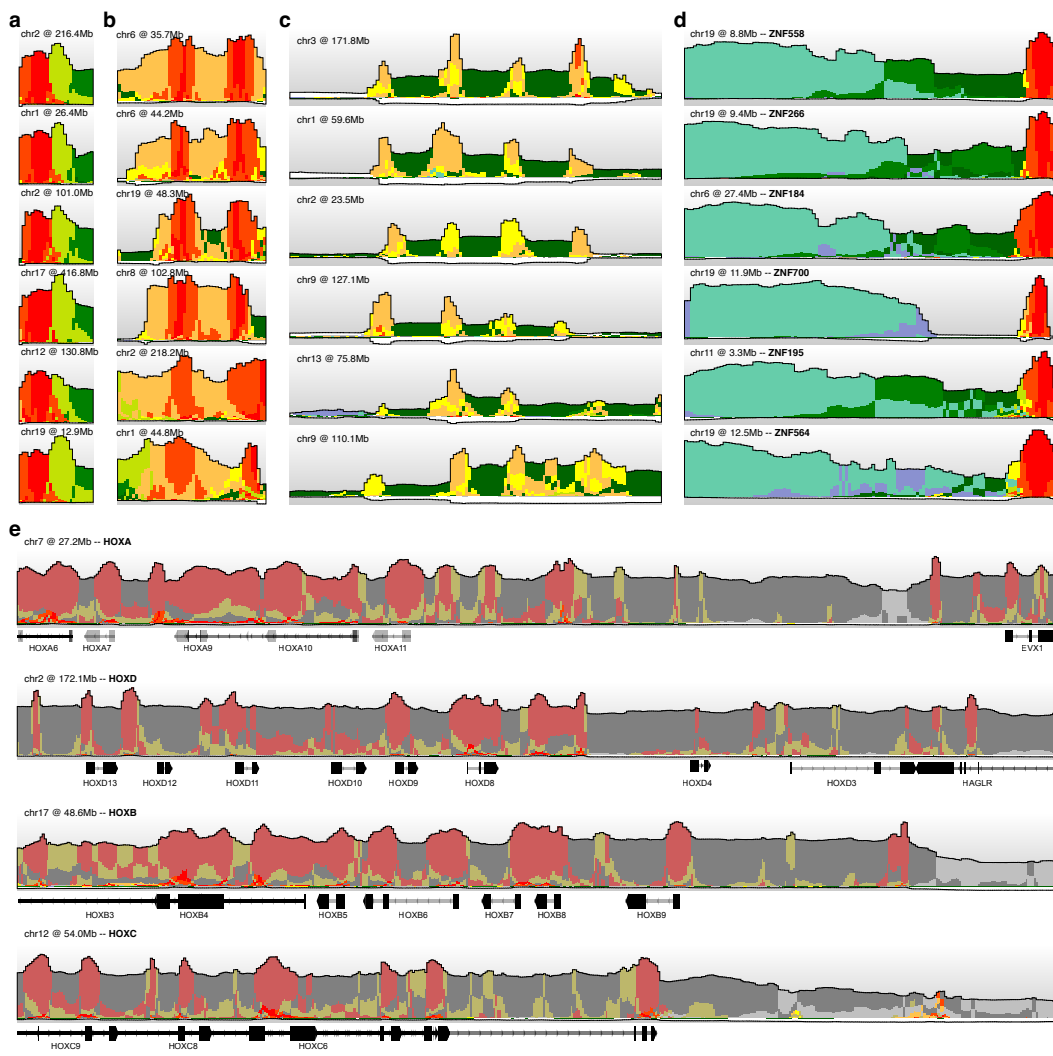

Supplement: Supplement 3 — Supp. Figure 3 – Similarity search results across genomic scales (a) a 5kb promoter-like region on human chromosome 2 and its 5 most similar regions elsewhere in the genome. (b) a 10kb region with strong regulatory signals on chromosome 6 and its top 5 similar regions. (c,d) two separate 10kb regions on chromosomes 3 and 19, highlighting similarity across loosely organized clusters of enhancers (c) and regions overlapping zinc-finger genes (d, gene names indicated). (e) A large 100kb region in the HOXA locus, with functionally highly related matches in other hox loci, namely HOXD, HOXB and HOXC. [file media-3.pdf]
